# Factors associated with post-pandemic acceptance of COVID-19 vaccines among students in three Nigerian universities

**DOI:** 10.1371/journal.pone.0312271

**Published:** 2024-12-04

**Authors:** Adaobi Uchenna Mosanya, Adaeze Ezekwelu, Ezinwanne Jane Ugochukwu, Blessing Onyinye Ukoha-Kalu

**Affiliations:** 1 Clinical Pharmacy and Pharmacy Management, Faculty of Pharmaceutical Sciences, University of Nigeria, Nsukka, Enugu State, Nigeria; 2 School of Medicine, University of Nottingham, Nottingham, England, United Kingdom; University of Rwanda College of Medicine and Health Sciences, RWANDA

## Abstract

**Background:**

The COVID-19 pandemic impacted the world in every aspect. Higher institutions were greatly affected because the outbreak disrupted the teaching and learning structure. Vaccines decrease the rate of infection and transmission of the virus, but the presence of some myths has led to hesitancy towards the vaccine.

**Objective:**

The purpose of the survey was to assess the knowledge, perception, and acceptance of the COVID-19 vaccine among undergraduate students in Enugu State, Nigeria.

**Methods:**

This was a cross-sectional study carried out among undergraduate students at the University of Nigeria Nsukka (UNN), the Institute of Management and Technology (IMT), and Enugu State University of Technology Enugu state (ESUT), Nigeria between March and November 2023. These institutions were chosen based on their large student populations, diverse academic offerings, and significant geographical coverage within the state. Data collection was done using a 26-item validated self-administered questionnaire. Statistical Product and Service Solutions (SPSS) version 25 with appropriate descriptive (frequency and percentage) and inferential statistics (Chi-square) were used to analyze the data.

**Results:**

1,143 completed questionnaires were obtained. The modal age range was 18–24 years accounting for 814 (71.2%) of the participants. A total of 577 (50.5%) participants demonstrated a good level of knowledge while 685 (59.9%) showed a positive perception of the COVID-19 vaccine. Gender (p = 0.010) and institution (p < 0.001) were associated with their perception of the COVID-19 vaccine. In addition, knowledge and perception of the COVID-19 vaccine were significantly associated with its acceptance at p = 0.038 and < 0.001, respectively.

**Conclusion:**

This study reveals that COVID-19 vaccine acceptance among university students in Enugu State, Nigeria, remains low despite moderate knowledge and generally positive attitudes, with perceptions playing a more significant role than knowledge. The findings highlight the need for educational interventions that not only provide accurate information but also actively address misconceptions. To improve vaccine uptake, public health campaigns should focus on shifting perceptions through culturally sensitive, institution-specific strategies.

## Introduction

COVID-19 was first recorded in December 2019 with the discovery of a cluster of patients suffering from pneumonia of unclear cause in Wuhan City, Hubei province of China. The severe acute respiratory syndrome coronavirus- 2 which has spread to more than 200 countries with more than 760 million reported cases and 6.9 million confirmed deaths [1] is a new strain from the family of coronaviruses (Coronaviruses) identified as the cause of these uncommon infections [2].

As of June 2023, over 13 billion vaccine doses have been administered [1]. Although WHO announced the end of the COVID-19 emergency phase in May 2023 [1], there is a continuous rise in the number of new cases globally [3]. This highlights the need for continuous vaccination. In reality, vaccine hesitancy is defined as “the delay in acceptance or refusal of vaccination despite the availability of vaccination services” [4] and is a significant issue in Africa today [5]. A good proportion of Nigerians just like in other countries, considered the vaccine safe and effective in the prevention and control of the pandemic [6], however, the acceptance rate ranges from 20–56% [7].

Tertiary institutions, globally, were highly affected by the COVID-19 pandemic [8–10] resulting in psychological distress, interruptions in learning, and anxiety. Previous studies assessing COVID-19 vaccine acceptance or hesitancy as well as the contributing factors were not specifically carried out among the students of tertiary institutions. There seems to be a mix in the population, introducing a high level of heterogeneity in the generated data. Some compelling factors were identified as reasons for vaccine hesitancy such as conspiracy theories, disbelief, uncertainty about its safety, etc, but none highlighted the factors peculiar to students of higher institutions. Enugu state is one of the states in Nigeria that has more than 30 tertiary institutions. Therefore, there is a need to assess the acceptance of the COVID-19 vaccine among students of tertiary institutions in Enugu state and associated factors. This would provide data specific to students of higher institutions and form the basis for generalization. Additionally, there is a potential to raise awareness and foster more positive attitudes toward vaccination. Identifying the factors that affect vaccine acceptance among this population would assist in designing and implementing a program aimed at improving vaccine uptake in Enugu State.

## Methods

### Study design

This was a cross-sectional study carried out among students of three higher institutions in Enugu state Nigeria. The ethical approval to carry out the study was granted by the Research Ethics Committee of the Faculty of Pharmaceutical Sciences, University of Nigeria with a reference number FPSRA/UNN/22/0044. During the study, no personal identifying data was collected to ensure the confidentiality of the responses. We obtained informed written consent from the study participants before enrolling them in the study.

### Study setting

The study was carried out at three higher institutions in Enugu State, Nigeria. These were the University of Nigeria Nsukka (UNN), the Institute of Management and Technology (IMT), and the Enugu State University of Technology Enugu state (ESUT), Nigeria between March and November 2023. These institutions were chosen based on their large student populations, diverse academic offerings, and significant geographical coverage within the state. We prioritized institutions with large and diverse student bodies to ensure that our sample would be representative of the broader student population in Enugu State. To capture a range of perspectives and experiences, we selected institutions that differ in their academic focus and type (a federal university, a state university, and a polytechnic). The selected institutions are strategically located in different parts of Enugu State, ensuring that the study captures data from students in various urban and rural settings.

### Eligibility criteria

The eligibility criteria for participation in the study were being an undergraduate student in the above higher institutions and granting informed written consent to take part.

### Sampling and sample size estimation

This study employed a non-probability purposive and convenience sampling technique to recruit the participants. With the assumption of a marginal error of not more than 2% with a 95% confidence level and a prevalence of COVID-19 vaccine acceptance of approximately 20%, the sample size was estimated as 1537 [11].

### Data collection instrument

A 26-item self-administered questionnaire was used for this study (S1 File). Content validation was carried out by three academic lecturers of the Department of Clinical Pharmacy and Pharmacy Management, Faculty of Pharmaceutical Sciences, University of Nigeria Nsukka. Section A of the questionnaire consists of eight items on the sociodemographic characteristics of the participants. Section B consists of eight questions that assess the knowledge of the participants regarding the COVID-19 vaccine with a “Yes” or “No” response option. Section C consists of eight perception items regarding the COVID-19 vaccine with a five-point Likert scale response from Strongly disagree- Strongly agree. Finally, two questions were used to assess the acceptance level of the participants towards the COVID-19 vaccine with a “Yes” or “No” or “I don’t know” response option. The questionnaire was distributed to the students in various settings such as their hostels, in their classrooms before lectures, or in public spaces within the institution’s premises.

### Data analysis

The completed questionnaires were coded and entered into Microsoft Excel. Thereafter, the raw data were cleaned and then exported into the Statistical Product and Service Solutions (SPSS) version 25 (IBM Corp., Armonk, N.Y., USA) for analysis. Frequencies and percentages were used to describe the analyzed data. Inferential statistics were done by computing the Chi-square test of the association of knowledge, perception, and acceptance scores and the demographic characteristics of the respondents, and the p-value was set as 0.05. After computing the knowledge and perception response scores, it was found that they were not normally distributed. Therefore, the median was used as the cut-off. The respondents with scores equal or more than the median score had good knowledge while those having scores equal or above the median were categorized as having a positive perception of the COVID-19 vaccine.

## Results

**Fig 1:**
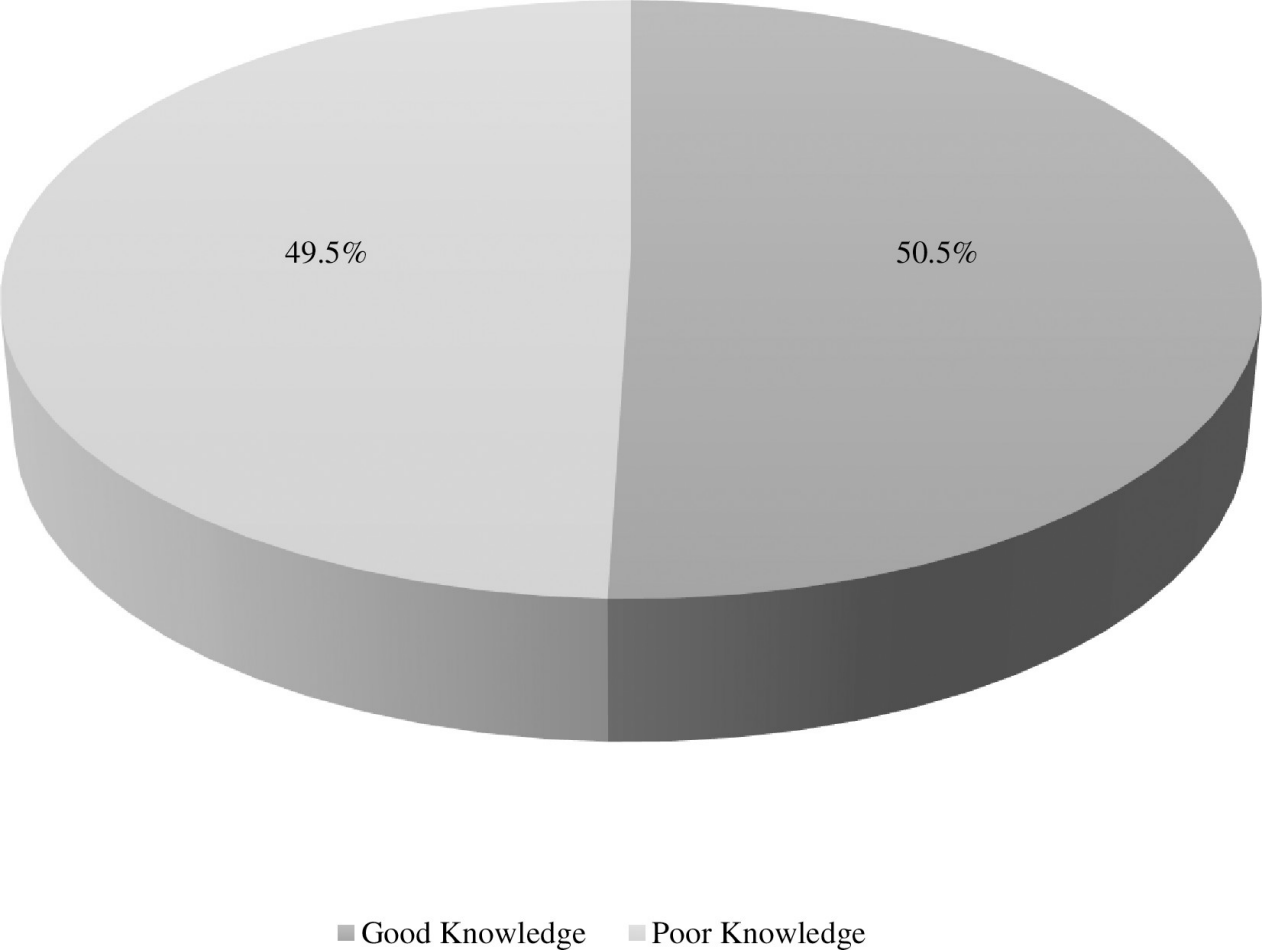
The level of knowledge of the COVID-19 vaccine.

In Table 1, a total number of 1,143 students completed the survey while the estimated sample size was 1537. Therefore, the response rate was 74%. Most of the participants 814 (71.2%) were within the age range of 18–24 years. A total of 610 (53.4%) of the students live off-campus. Most of the students, 1054 (92.2%) gained admission through the post-Unified Tertiary Matriculation Examination (UTME). A total of 577 (50.5%) of the respondents had a good knowledge of the COVID-19 vaccine (Fig 1). In Table 2, the statement “Is it dangerous to use an overdose of COVID-19 vaccine?” had the highest percentage of “Yes” answers 1031 (90.2%). Also, 585 (51.2%) of the respondents stated “No” to the statement “Is COVID-19 vaccine suitable for pregnant women?” From the study, 94.0% had not taken the COVID-19 vaccine. The level of acceptance was very low 69 (6%) and only about 5% of those who had taken it would recommend it to family or friends. In Table 3, almost half of 478 (41.9%) agreed that there are “Scary information about the vaccine is rampant on social media”. Also, 454 (39.7%) of the respondents agreed that “COVID-19 vaccine is safe and essential for use”. Table 4 shows the proportion of study participants who had good knowledge, good perception, and acceptance of the COVID-19 vaccine. Tables 5 and 6 shows that there was no significant association (*p* >0.05) between the sociodemographic variables and the knowledge about the COVID-19 vaccine. However, the perception of the respondents towards the COVID-19 vaccine was significantly associated with gender (*p* = 0.010) and institution (*p* <0.001). In addition, knowledge and perception of the COVID-19 vaccine were significantly associated with its acceptance at p = 0.038 and < 0.001 respectively. Table 7 shows the logistic regression results. Only knowledge and perception regarding the vaccine were significantly associated with acceptance of the COVID-19 vaccine after the chi-square analysis. Only these two variables were entered as covariates in the logistics analysis to identify the predictors. They both emerged as predictors after adjusting for the confounding effects of both. Those with good knowledge have less odds of accepting the vaccine than those with poor knowledge. However, those with good perception have four times the odds of accepting the vaccine than those with poor perception.

**Table 1:** Demographic information of respondents (N = 1,143).

Demographics		Number	Percentage
**Age (Years)**	Less than 18	51	4.5
	18–24	814	71.2
	25–31	269	23.4
	32–38	8	0.7
	Above 38	3	0.3
**Residence**	Hostel	533	46.6
	Off campus	610	53.4
**Gender**	Male	318	27.8
	Female	825	72.2
**Marital status**	Single	1074	94.0
	Married	69	6.0
**Mode of admission**	Post-UTME	1054	92.2
	Direct- Entry	89	7.8
**Religion**	Christianity	1114	97.5
	Islam	19	1.7
	African traditional religion	10	0.9
**Ethnic group**	Igbo	1079	94.4
	Yoruba	25	2.2
	Hausa	10	.9
	Cameroon	3	.3
	Annang	4	.3
	Ghanaian	2	.2
	Ibibio	7	.6
	Edo	4	.3
	Idoma	8	.7
	others	1	.1
**Higher institution**	University of Nigeria	538	47.1
	Institute of Management and Technology	279	24.4
	Enugu State University of Science and Technology	326	28.5

**Table 2:** Knowledge and acceptance of COVID-19 vaccine.

S/N	Statements	No		Yes	
		Number	Percentage	Number	Percentage
1	Is the COVID-19 vaccine given by only injection	228	19.9	915	80.1
2	Can the COVID-19 vaccine protect the receiver from getting COVID-19 infection?	291	25.5	852	74.5
3	Is it dangerous to use an overdose of COVID-19 vaccine?	112	9.8	1031	90.2
4	Does COVID-19 vaccination cause allergic reactions?	294	25.7	849	74.3
5	Does vaccination increase autoimmune disease?	365	31.9	778	68.1
6	Is the COVID-19 vaccine suitable for pregnant women?	585	51.2	558	48.8
7	Can everyone including children receive the COVID-19 vaccine?	330	28.9	813	71.1
8	Do COVID-19 vaccines have side effects?	225	19.7	918	80.3
9	Have you taken the vaccine?	1074	94.0	69	6.0
10	If yes, do you encourage your family/friends/relatives to get vaccinated?	8	0.7	55	4.8

**Table 3:** Perception towards COVID-19 vaccine.

S/N	Statements	Strongly Disagree		Disagree		Neutral		Agree		Strongly Agree	
		n	%	n	%	n	%	n	%	n	%
1	COVID-19 vaccine is safe	48	4.2	85	7.4	371	32.5	454	39.7	185	16.2
2	COVID-19 Vaccine is essential for use	66	5.8	125	10.9	347	30.4	454	39.7	151	13.2
3	COVID-19 may cause infection	128	11.2	406	35.5	362	31.7	160	14.0	87	7.6
4	COVID-19 vaccine may not be effective	89	7.8	188	16.4	341	29.8	415	36.3	110	9.6
5	It is not possible to reduce the incidence of COVID-19 without vaccination	156	13.6	247	21.6	241	21.1	330	28.9	169	14.8
6	Scary information about the vaccine is rampant on social media	22	1.9	49	4.3	139	12.2	478	41.8	455	39.8
7	COVID-19 vaccine can protect me from getting infected	117	10.2	150	13.1	394	34.5	330	28.9	152	13.3
8	I am afraid to take the COVID-19 vaccine	89	7.8	100	8.7	340	29.7	336	29.4	278	24.3

**Table 4:** Good knowledge, good perception and acceptance of COVID-19 vaccine.

Characteristics		Total (1143)	Good knowledge (577)	Good Perception (685)	Acceptance (69)
Age (Years)	Less than 18	51(4.5)	27(52.9)	29(56.9)	3(5.9)
	18–24	814(71.2)	415(51.0)	481(59.1)	47(5.8)
	25–31	267(23.4)	129(48.3)	166(62.2)	16(6.0)
	32–38	8(0.7)	5(62.5)	6(75)	2(25)
	Above 38	3(0.3)	1(33.3)	3(100)	1(33.3)
Residence	Hostel	533(46.6)	270(50.7)	321(60.2)	27(5.1)
	Off campus	610(53.4)	307(50.3)	364(59.7)	42(6.9)
Gender	Male	318(27.8)	155(48.7)	171(53.8)	21(6.6)
	Female	825(72.2)	422(51.2)	514(62.3)	48(5.8)
Marital Status	Single	1074(94)	545(50.7)	642(59.8)	65(6.1)
	Married	69(6.0)	32(46.4)	43(62.3)	4(5.8)
Mode of Admission	JAMB	1054(92.2)	540(51.2)	623(59.1)	65(6.2)
	DE	89(7.8)	37(41.6)	62(69.7)	4(4.5)
Religion	Christianity	1114(97.5)	564(50.6)	666(59.8)	65(5.8)
	Islam	19(1.7)	10(52.6)	14(73.7)	3(15.8)
	ATR	10(0.9)	3(30.0)	5(50.0)	1(10.0)
Ethnicity	Igbo	1079(94.4)	548(50.8)	651(60.3)	62(5.7)
	Yoruba	25(2.2)	11(44.0)	14(56.0)	1(4.0)
	Hausa	10 (0.9)	3(30.0)	5(50.0)	2(20.0)
	Cameron	3(0.3)	1(33.3)	0 (0.0)	0(0.0)
	Annang	4(0.3)	2(50.0)	3(75.0)	0(0.0)
	Ghanaian	2(0.2)	2(100)	1(50.0)	1(50.0)
	Ibibio	7(0.6)	2(28.6)	6(85.7)	1(14.3)
	Edo	4(0.3)	4(100)	1(25.0)	1(25.0)
	Idoma	8(0.7)	4(50.0)	3(37.5)	1(12.5)
	Others	1(0.1)	0(0.0)	3(100)	0(0.0)
Higher Institution	UNN	538(47.1)	285(53.0)	293(54.5)	29(5.4)
	IMT	279(24.4)	128(45.9)	162(58.1)	25(9.0)
	ESUT	326(28.5)	164(50.3)	230(70.6)	15(4.6)
Taken the Vaccine	No	1074(94.0)	551 (51.3)	627(58.4)	NA
	Yes	69(6.0)	26 (37.7)	58(84.1)	NA

**Table 5:** Association between Sociodemographic and level of knowledge, perception, and acceptance of the respondents.

Characteristics		Knowledge (A) N (%)		χ2	Perception (B) N (%)		χ2	Acceptance (C) N (%)		χ2
		Good knowledge	Poor Knowledge		Good Perception	Poor Perception		Acceptance	Rejection	
**Age (Years)** *P*(A) = .823 *P*(B) = .439 *P*(C) = .058	Less than 18	27 (2.4)	24(2.1)	1.522	29(2.5)	22(1.9)	3.760	3(0.3)	48(4.2)	9.115
	18–24	415(36.3)	399(34.9)		481(42.1)	333(29.1)		47(4.1)	767(67.1)	
	25–31	129(11.3)	138(12.1)		166(14.5)	101(8.8)		16(23.2)	251(22.0)	
	32–38	5(0.4)	3(0.3)		6(0.5)	2(0.2)		2(0.2)	6(0.5)	
	Above 38	1(0.1)	2(0.2)		3(0.3)	0(0.0)		1(0.1)	2(0.2)	
**Residence** *P*(A) = .959* P(B) = .897* P(C) = .244*	Hostel	270(23.6)	263(23.0)	0.003*	321(28.1)	212(18.5)	0.017*	27(2.4)	506(44.3)	**1.355** *
	Off-campus	307(26.9)	303(26.5)		364(31.8)	246(21.5)		42(3.7)	568(49.7)	
**Gender** *P*(A) = .507* P(B) = .010* P(C) = .718*	Male	155(26.9)	163(28.8)	0.441*	171(15.0)	147(12.9)	**6.603** *	21(1.8)	297(26.0)	0.130*
	Female	422(36.9)	403(35.3)		514(45.0)	311(27.2)		48(4.2)	777(68.0)	
**Marital Status** *P*(A) = .562* P(B) = .771* P(C) = 1.000*	Single	545(47.7)	529(46.3)	0.336*	642(56.2)	432(37.8)	0.085*	65(5.7)	1009(88.3)	<0.001*
	Married	32(2.8)	37(3.2)		43(3.8)	26(2.3)		4(0.3)	65(5.7)	

*Continuity correction value for 2 X 2 tables P(A). P(B) and P(C) stands for the p-value for the knowledge, perception, and acceptance of the COVID-19 vaccine chi-square association with the respective variables.

**Table 6:** Association between sociodemographic and level of knowledge, perception and acceptance of the respondents.

Characteristics		Knowledge (A) N (%)		χ2	Perception (B) N (%)		χ2	Acceptance (C) N (%)		χ2
		Good knowledge	Poor Knowledge		Good Perception	Poor Perception		Acceptance	Rejection	
**Mode of Admission** *P*(A) = .101* P(B) = .066* P(C) = .686*	JAMB	540(47.2)	514(45.0)	2.690*	623(54.5)	431(37.7)	3.380*	65(5.7)	989(86.5)	.164*
	DE	37(3.2)	52(4.5)		62(5.4)	27(2.4)		4(0.3)	85(7.4)	
**Religion** *P*(A) = .423 *P*(B) = .383 *P*(C) = .170	Christianity	564(49.8)	550(48.1)	1.723	666(58.3)	448(39.8)	1.917	65(5.7)	1049(91.8)	3.543
	Islam	10(0.9)	9(0.8)		14(1.2)	5(0.4)		3(0.3)	16(1.4)	
	ATR	3(0.3)	7(0.6)		5(0.4)	5(0.4)		1(0.1)	9(0.8)	
**Ethnicity** *P*(A) = .294 *P*(B) = .219 *P*(C) = .089	Igbo	548(47.9)	531(46.5)	10.742	651(57.0)	428(37.4)	11.907	62(89.0)	1017(89.0)	15.074
	Yoruba	11(1.0)	14(1.2)		14(1.2)	11(1.0)		1(0.1)	24(2.1)	
	Hausa	3(0.3)	7(0.6)		5(0.4)	5(0.4)		2(0.2)	8(0.7)	
	Annang	2(0.2)	2(0.2)		3(0.3)	1(0.1)		0(0.0)	4(0.3)	
	Ghanaian	2(0.2)	0(0.0)		1(0.1)	1(0.1)		1(0.1)	1(0.1)	
	Ibibio	2(0.2)	5(0.4)		6(0.5)	1(0.1)		1(0.1)	6(0.5)	
	Edo	4(0.3)	0(0.0)		1(0.1)	3(0.3)		1(0.1)	3(0.3)	
	Idoma	4(0.3)	4(0.3)		3(0.3)	5(0.4)		1(0.1)	7(0.6)	
	Others	3(0.3)	3(0.3)		3(0.3)	2(0.2)		1(0.1)	5(0.5)	
**Higher Institution** *P*(A) = .157 ***P*(B) = < .001** *P*(C) = .055 **Taken the Vaccine** *P(A) =* < 0.038* P(B) = < 0.001*	UNN	285(24.9)	253(22.1)	3.706	293(25.6)	245(21.4)	**22.422**	29(2.5)	509(44.5)	5.785
	IMT	128(11.2)	151(13.2)		162(14.2)	117(10.2)		25(2.2)	254(22.2)	
	ESUT	164(14.3)	162(14.2)		230(20.1)	96(8.4)		15(1.3)	311(27.2)	
	No Yes	551 (48.2) 26 (2.3)	523 (45.8) 43 (3.8)	4.283*	627(54.9) 58(5.1)	447(39.1) 11(1.0)	16.749*	NA	NA	

NA: Not applicable because taking the vaccine was interpreted as acceptance

**Table 7:** Logistics regression COVID-19 vaccine predictors.

Variables	Acceptance of COVID-19 vaccine n (%)	COR (95% CI)	AOR (95% CI)
Knowledge			
Poor (566)	43 (62.3)	Reference	Reference
Good (577)	26 (37.7)	0.574 (0.348–0.948) *	0.595 (0.359–0.986) *
Perception			
Poor (458)	11 (15.9)	Reference	Reference
Good (685)	58 (84.1)	3.759 (1.951–7.243) **	3.692 (1.914–7.119) **

*p< 0.05

** p < 0.001

## Discussion

This study’s findings evaluated the level of knowledge, perception, and acceptance of the COVID-19 vaccine among students in three tertiary institutions in Enugu state, Nigeria. About half of the students had a good knowledge of the COVID-19 vaccine. This is comparable to a study by Jiang et al showing good knowledge about the COVID-19 vaccine among the participants [12]. One may assume that such an observation was because undergraduate students are expected to be exposed to certain information inaccessible to the other less privileged members of the general population. This current study identified a significant association between good knowledge and vaccine acceptance. A significantly lower proportion of those with good knowledge who accepted the vaccine than those with poor knowledge. The logistics regression showed that those with good knowledge were less likely to accept the vaccine than those with poor knowledge. Perhaps, knowledge about the vaccine is not necessary to accept the vaccine.

On the other hand, good perception was a highly predictive factor for vaccine acceptance. Perhaps, it is sufficient for the population to have a good perception of the vaccine to accept vaccination. This is in line with the outcomes from a scoping review done on COVID-19 vaccine hesitancy in Africa. It was found that among other factors that facilitate the acceptability of the COVID-19 vaccine were knowledge and positive perception about the vaccine [5].

In this study, gender and institution of the study participants were significantly associated with their acceptance towards the COVID-19 vaccine with females showing a higher proportion with good perception as compared to the males. In addition, perception was significantly associated with acceptance of the vaccine. This would serve as a guide in targeting the males through campaigns aimed to increase vaccine uptake. However, it is interesting to note that a similar study in Ontario provided a contrary conclusion. Females were found to have poor perception of the vaccine and higher hesitancy compared to the males [13]. On the other hand, the institutions with low proportions of the participants having good perceptions would be targeted for improved vaccine uptake.

The findings from this study should be interpreted in the light of its strengths and limitations. One of its strengths was the focus on only students of tertiary institutions unlike other previous studies in Nigeria which included a heterogeneous population [14–16]. This would allow highlighting implications for policies geared towards increased COVID-19 vaccine acceptance and uptake. However, the limitation of using a questionnaire that was only face-validated would pose a problem in the generalizability of the findings.

Nevertheless, conclusions can be made regarding the acceptance of the COVID-19 vaccine among students of tertiary institutions in Enugu State, Nigeria. Firstly, there was a moderate level of good knowledge about the vaccine. Secondly, the acceptance level was very poor. Thirdly, perception and knowledge were significant predictors of vaccine acceptance. Therefore, implications for future research should be done using a content-validated questionnaire in addition to face validation. Also, to improve the generalizability of the findings, efforts should be directed to increasing the response rate. In addition, the study design may adopt a case-control method for more robust conclusions.

An implication for policy is a targeted campaign to improve the perception concerning the COVID-19 vaccine in all tertiary institutions which would increase the vaccine acceptance and uptake. The campaigns can be in the form of seminars, workshops, etc. organized specifically for students of tertiary institutions in Nigeria.

## Conclusion

This study provides valuable insights into the post-pandemic acceptance of COVID-19 vaccines among university students in Enugu State, Nigeria. Despite a moderate level of knowledge and generally positive perceptions towards the vaccine, acceptance rates remained low, influenced significantly by the students’ perceptions rather than their knowledge alone. The findings underscore the importance of targeted educational interventions that not only disseminate accurate information but also actively address misconceptions and enhance positive perceptions. This approach could be instrumental in improving vaccine uptake among this demographic. Our study highlights the critical role of perception in vaccine acceptance, suggesting that future public health campaigns should prioritize shifting perceptions through culturally sensitive, institution-specific strategies. These findings contribute to the ongoing dialogue on vaccine hesitancy and suggest actionable steps for increasing vaccine acceptance in similar contexts.

### Policy implication

There is a need to ensure that information concerning the COVID-19 vaccine should be spread to all institutions via health promotion campaigns and public health talks. The heads of tertiary institutions should organize seminars, and workshops on vaccination acceptance and recommendations to all students.

## Supporting information

S1 File Study questionnaire. (PDF)
