# Supplementary material for: Factors associated with post-pandemic acceptance of COVID-19 vaccines among students in three Nigerian universities
Source: PLoS One. 2024 Dec 4;19(12):e0312271. doi: 10.1371/journal.pone.0312271 (PMC11616807; doi:10.1371/journal.pone.0312271)
Supplement: S1 File — (PDF) [file pone.0312271.s001.pdf]

S1 File

Study questionnaire

SECTION A: Socio-Demography

1. Age (years): <18 [ ] 18-24 [ ] 25-31 [ ] 32-38 [ ] Above 38 [ ]
2. Residence: Hostel [ ] Off campus
3. Sex: Male [ ] Female [ ]
4. Marital status: Single [ ] Married [ ]
5. Mode of admission: JAMB [ ] DE [ ]
6. Religion: Christianity [ ] Islam [ ] African Traditional Religion [ ] Others (please specify)……………..
7. Ethnicity: Igbo [ ] Yoruba [ ] Hausa [ ] Others (please specify) ……………………
8. Higher Institution: University of Nigeria, Nsukka [ ] Institute of Management and Technology, Enugu. [ ] Enugu State University of Science and Technology. [ ]

SECTION B: Knowledge about COVID-19 vaccine

	YES	NO
1. Is COVID-19 Vaccine given only by injection?		
2. Can COVID-19 Vaccine protect the receiver from getting COVID-19 infections?		
3. Is it dangerous to use an overdose of COVID-19 vaccine?		
4. Can COVID-19 vaccination give allergic reactions?		
5. Does COVID-19 vaccination increase autoimmune diseases?		
6. Is COVID-19 Vaccine suitable for pregnant women?		
7. Can everyone including children receive the COVID-19 vaccine?		
8. Do COVID-19 vaccines have side effects?		

SECTION C: Perception towards COVID-19 vaccine

SD=Strongly Agree D=Disagree N=Neutral A=Agree SA=Strongly Agree

	SA	A	N	D	SD
1. COVID-19 vaccine is safe					
2. COVID-19 vaccine is essential for us					
3. COVID-19 vaccine may cause infection					
4. COVID-19 vaccine may not be effective					
5. It is not possible to reduce the incidence of COVID-19 without vaccination					
6. Scary information about the vaccine are rampart on social media					
7. COVID-19 vaccine can protect me from getting infected					
8. I am afraid to take the COVID-19 vaccine					

SECTION D: Acceptance of COVID-19 vaccine

1. Have you taken the vaccine? Yes [ ] No [ ]
2. If your response to question 1 is yes, do you encourage your family/ friends/ relatives to get vaccinated? Yes [ ] No [ ]
