# Supplementary material for: Factors associated with post-pandemic acceptance of COVID-19 vaccines among students in three Nigerian universities
Source: PLoS One. 2024 Dec 4;19(12):e0312271. doi: 10.1371/journal.pone.0312271 (PMC11616807; doi:10.1371/journal.pone.0312271)
Supplement: Attachment — Submitted filename: PONE-D-24-14109_.pdf [file pone.0312271.s002.pdf]

PLOS ONE

Factors associated with post-pandemic acceptance of COVID-19 vaccines among students in three Nigerian Universities

--Manuscript Draft--

**Manuscript Number:**	PONE-D-24-14109
**Article Type:**	Research Article
**Full Title:**	Factors associated with post-pandemic acceptance of COVID-19 vaccines among students in three Nigerian Universities
**Short Title:**	Post-pandemic acceptance of COVID-19
**Corresponding Author:**	Blessing Onyinye Ukoha-kalu, B.Pharm, M.Pharm, Ph.D<br>University of Nottingham School of Medicine<br>Nottingham, --- Select One --- UNITED KINGDOM
**Keywords:**	COVID-19 Vaccine; Vaccine acceptance; vaccine hesitancy; students; knowledge
**Abstract:**	Background<br><br>The COVID-19 pandemic impacted the world in every aspect. Higher institutions were affected because the teaching and learning structure were disrupted by the outbreak. Vaccine decreases the rate of infection and transmission of the virus, but the presence of some myths has led to hesitancy towards the vaccine.<br><br>Objective<br><br>The purpose of the survey was to assess the knowledge, perception, and acceptance of the COVID-19 vaccine among undergraduate students in Enugu State, Nigeria.<br><br>Methods<br><br>This was a cross-sectional study in which responses were collected among students of three higher institutions. Data collection was done with a 26-item validated self-administered questionnaire. The data were described using frequencies and percentages. Inferences were made using the Chi-square test of association between the sociodemographic data and the knowledge, perception, and acceptance of the COVID-19 Vaccine, respectively.<br><br>Results<br><br>1,143 completed questionnaires were obtained. The modal age range was 18-24 years accounting for 814 (71.2%) of the participants. About half of them 577 (50.5%) demonstrated a good level of knowledge of the COVID-19 vaccine while 685 (59.9%) possessed a positive perception. Gender ($p = 0.010$) and institution ($p < 0.001$) were associated with their perception regarding the COVID-19 vaccine. In addition, knowledge and perception of the COVID-19 vaccine were significantly associated with its acceptance at $p = 0.038$ and < 0.001, respectively.<br><br>Conclusion<br><br>Undergraduate students in Enugu had good knowledge and positive perceptions regarding the COVID-19 vaccine. There was a significant association between the students' gender and the institution with their perception of the COVID-19 vaccine. On the other hand, acceptance of the COVID-19 vaccine by the students was associated with their knowledge and perception of the COVID-19 vaccine.
**Order of Authors:**	Adaobi Uchenna Mosanya
	Adaeze Ezekwelu, BPharm, MPharm
	Ezinwanne Jane Ugochukwu, BPharm, MPharm, PhD
	Blessing Onyinye Ukoha-kalu, B.Pharm, M.Pharm, Ph.D

Powered by Editorial Manager® and ProduXion Manager® from Aries Systems Corporation

Additional Information:	
**Question**	**Response**
**Financial Disclosure**<br><br>Enter a financial disclosure statement that describes the sources of funding for the work included in this submission. Review the submission guidelines for detailed requirements. View published research articles from *PLOS ONE* for specific examples.<br><br>This statement is required for submission and **will appear in the published article** if the submission is accepted. Please make sure it is accurate.<br><br>**Funded studies**<br>Enter a statement with the following details:<br>• Initials of the authors who received each award<br>• Grant numbers awarded to each author<br>• The full name of each funder<br>• URL of each funder website<br>• Did the sponsors or funders play any role in the study design, data collection and analysis, decision to publish, or preparation of the manuscript?<br><br>Did you receive funding for this work?	The author(s) received no specific funding for this work.
**Competing Interests**<br><br>Use the instructions below to enter a competing interest statement for this submission. On behalf of all authors, disclose any competing interests that could be perceived to bias this work—acknowledging all financial support and any other relevant financial or non-financial competing interests.<br><br>This statement is required for submission and **will appear in the published article** if the submission is accepted. Please make sure it is accurate and that any funding sources listed in your Funding Information	The authors have no competing interests relevant to this article's content.

later in the submission form are also declared in your Financial Disclosure statement.<br><br>View published research articles from *PLOS ONE* for specific examples.<br><br>**NO authors have competing interests**<br><br>Enter: *The authors have declared that no competing interests exist.*<br><br>**Authors with competing interests**<br><br>Enter competing interest details beginning with this statement:<br><br>*I have read the journal's policy and the authors of this manuscript have the following competing interests: [insert competing interests here]*<br><br>* typeset	
**Ethics Statement**<br><br>Enter an ethics statement for this submission. This statement is required if the study involved:<br><br>• Human participants<br>• Human specimens or tissue<br>• Vertebrate animals or cephalopods<br>• Vertebrate embryos or tissues<br>• Field research<br><br>Write "N/A" if the submission does not require an ethics statement.<br><br>General guidance is provided below. Consult the submission guidelines for detailed instructions. **Make sure that all information entered here is included in the Methods section of the manuscript.**	The ethical approval to carry out the study was granted by the Research Ethics Committee of the Faculty of Pharmaceutical Sciences, University of Nigeria. The Reference number of the approval letter was FPSRA/UNN/22/0044. Through the survey, no personal identifying data was collected to ensure the confidentiality of the responses. The respondents granted their informed consent before participation.

**Format for specific study types**<br><br>**Human Subject Research (involving human participants and/or tissue)**<br>• Give the name of the institutional review board or ethics committee that approved the study<br>• Include the approval number and/or a statement indicating approval of this research<br>• Indicate the form of consent obtained (written/oral) or the reason that consent was not obtained (e.g. the data were analyzed anonymously)<br><br>**Animal Research (involving vertebrate animals, embryos or tissues)**<br>• Provide the name of the Institutional Animal Care and Use Committee (IACUC) or other relevant ethics board that reviewed the study protocol, and indicate whether they approved this research or granted a formal waiver of ethical approval<br>• Include an approval number if one was obtained<br>• If the study involved *non-human primates*, add *additional details* about animal welfare and steps taken to ameliorate suffering<br>• If anesthesia, euthanasia, or any kind of animal sacrifice is part of the study, include briefly which substances and/or methods were applied<br><br>**Field Research**<br><br>Include the following details if this study involves the collection of plant, animal, or other materials from a natural setting:<br>• Field permit number<br>• Name of the institution or relevant body that granted permission	
**Data Availability**<br><br>Authors are required to make all data underlying the findings described fully available, without restriction, and from the time of publication. PLOS allows rare exceptions to address legal and ethical concerns. See the PLOS Data Policy and FAQ for detailed information.	Yes - all data are fully available without restriction

A Data Availability Statement describing where the data can be found is required at submission. Your answers to this question constitute the Data Availability Statement and **will be published in the article**, if accepted.<br><br>**Important:** Stating 'data available on request from the author' is not sufficient. If your data are only available upon request, select 'No' for the first question and explain your exceptional situation in the text box.<br><br>Do the authors confirm that all data underlying the findings described in their manuscript are fully available without restriction?	
**Describe where the data may be found in full sentences. If you are copying our sample text, replace any instances of XXX with the appropriate details.**<br><br>• If the data are **held or will be held in a public repository**, include URLs, accession numbers or DOIs. If this information will only be available after acceptance, indicate this by ticking the box below. For example: *All XXX files are available from the XXX database (accession number(s) XXX, XXX.*).<br>• If the data are all contained **within the manuscript and/or Supporting Information files**, enter the following: *All relevant data are within the manuscript and its Supporting Information files.*<br>• If neither of these applies but you are able to provide **details of access elsewhere**, with or without limitations, please do so. For example:<br><br>*Data cannot be shared publicly because of [XXX]. Data are available from the XXX Institutional Data Access / Ethics Committee (contact via XXX) for researchers who meet the criteria for access to confidential data.*<br><br>*The data underlying the results presented in the study are available from (include the name of the third party*	All relevant data are within the manuscript and its Supporting Information files.

and contact information or URL).<br>• This text is appropriate if the data are owned by a third party and authors do not have permission to share the data.<br><br>* typeset	
Additional data availability information:	

Powered by Editorial Manager® and ProduXion Manager® from Aries Systems Corporation

Manuscript
Click here to access/download;Manuscript;Manuscript.docx

Factors associated with post-pandemic acceptance of COVID-19 vaccines among students in three Nigerian Universities

Adaobi Uchenna Mosanya[1], Adaeze Ezekwelu[1], Ezinwanne Jane Ugochukwu[1], Blessing Onyinye Ukoha-Kalu[*]

[1]Clinical Pharmacy and Pharmacy Management, Faculty of Pharmaceutical Sciences, University of Nigeria Nsukka Enugu State, Nigeria.
[2]School of Medicine, University of Nottingham, Nottingham, England, United Kingdom

*Corresponding author: blessing.ukoha-kalu@nottingham.ac.uk

ABSTRACT

Background: The COVID-19 pandemic impacted the world in every aspect. Higher institutions were affected because the teaching and learning structure were disrupted by the outbreak. Vaccine decreases the rate of infection and transmission of the virus, but the presence of some myths has led to hesitancy towards the vaccine.

Objective: The purpose of the survey was to assess the knowledge, perception, and acceptance of the COVID-19 vaccine among undergraduate students in Enugu State, Nigeria.

Methods: This was a cross-sectional study in which responses were collected among students of three higher institutions. Data collection was done with a 26-item validated self-administered questionnaire. The data were described using frequencies and percentages. Inferences were made using the Chi-square test of association between the sociodemographic data and the knowledge, perception, and acceptance of the COVID-19 Vaccine, respectively.

Results: 1,143 completed questionnaires were obtained. The modal age range was 18-24 years accounting for 814 (71.2%) of the participants. About half of them 577 (50.5%) demonstrated a good level of knowledge of the COVID-19 vaccine while 685 (59.9%) possessed a positive

perception. Gender ($p = 0.010$) and institution ($p < 0.001$) were associated with their perception regarding the COVID-19 vaccine. In addition, knowledge and perception of the COVID-19 vaccine were significantly associated with its acceptance at $p = 0.038$ and < 0.001, respectively.

Conclusion: Undergraduate students in Enugu had good knowledge and positive perceptions regarding the COVID-19 vaccine. There was a significant association between the students' gender and the institution with their perception of the COVID-19 vaccine. On the other hand, acceptance of the COVID-19 vaccine by the students was associated with their knowledge and perception of the COVID-19 vaccine.

Keywords: COVID-19 Vaccine; Vaccine acceptance; Vaccine hesitancy; Students; Knowledge

INTRODUCTION

COVID-19 began in December 2019 with the discovery of a cluster of patients suffering from pneumonia of unclear cause in Wuhan City, Hubei province of China. The coronavirus has now spread to more than 200 countries with more than 10 million reported cases and 508,000 confirmed deaths[1]. Severe Acute Respiratory Syndrome Coronavirus- 2 is a new strain from the family of coronaviruses (Coronaviruses) that was rapidly identified as the cause of these uncommon infections[2]. Patients with non-communicable diseases like diabetes mellitus or hypertension who also contracted the COVID-19 pandemic were found to have grim outcomes for the viral infection[3]. The World Health Organization reported that as of 29th June 2021, COVID-19 had killed over 4 million people (about twice the population of New Mexico) and infected over 180 million people worldwide[4]. According to the Nigeria Center for Disease Control and Prevention (2020), the Federal Ministry of Health in Nigeria verified the country's first COVID-19 case in February 2020. Between January 2020 and May 2023, Nigeria reported 266,675 confirmed cases

of COVID-19 with 3,155 deaths. As of March 2023, 116,606,863 vaccine doses had been administered[5]. Nigeria received its first shipment of COVID-19 vaccine in March 2021, thanks to UNICEF and the COVID-19 Vaccines Global Access (COVAX) initiative. The elderly, those with NCDs (non-communicable diseases), and medical professionals received the vaccine despite its limited availability at the time.

A significant issue in Africa today is vaccine hesitancy[6]. Vaccine hesitancy is defined as "the delay in acceptance or refusal of vaccination despite the availability of vaccination services"[7]. Misinformation and vaccine anxiety are major obstacles to coverage and community immunity in many nations[8]. Some studies have shown that students and health workers are quite hesitant to get vaccinated, especially in Africa[9,10] . The possibility of getting infected after vaccination, unfavourable side effects, and diminished efficacy are a few of the variables causing vaccine hesitancy[11–13]. A good percentage of Nigerians were ready to receive the vaccine during a survey before its introduction to the country[14]. However, most people in some northern Nigerian regions (99.5%) were well-informed about the vaccine, but only 29% of them accepted it[15]. The low vaccination rate in the North may be caused by records of low polio vaccine acceptance because of beliefs that women are at risk of HIV infection or infertility[16]. Age, gender, and educational level have been found to have a significant statistical relationship with acceptance of COVID-19 vaccine[17,18]. Tertiary institutions were highly affected by the COVID-19 pandemic. Nigerians just like in other countries, considered the vaccine to provide an effective control of the pandemic. The youth are seen as the future of every country, and they make up about 17% of the Nigerian population. Additionally, university students have the potential to raise awareness of certain issues related to public health and foster more positive attitudes toward vaccination[19]. Therefore, there was a need to assess the knowledge, perception and acceptance of the COVID-19 vaccine among university students in Enugu state, Nigeria.

METHODS

Study design

This was a cross-sectional study carried out among students of three higher institutions. The ethical approval to carry out the study was granted by the Research Ethics Committee of the Faculty of Pharmaceutical Sciences, University of Nigeria. The Reference number of the approval letter was FPSRA/UNN/22/0044. Through the survey, no personal identifying data was collected to ensure the confidentiality of the responses. The respondents granted their informed consent before participation.

Study setting

The survey took place in three higher institutions in Enugu State, Nigeria. They include the University of Nigeria Nsukka (UNN), the Institute of Management and Technology (IMT), and Enugu State University of Technology Enugu state (ESUT), Nigeria between March and November 2023. These higher institutions were selected because they admit a large population of students yearly.

Participants

The inclusion criteria for participation in the study were being an undergraduate student in the above higher institutions and granting informed free consent by the participants. The exclusion criteria were attending other higher institutions and unwillingness to participate in the survey.

Sampling and sample size estimation

Non-probability purposive and convenience sampling technique was used to obtain the participants. With the assumption of a marginal error of not more than 2% with a 95% confidence level and a prevalence of COVID-19 vaccine acceptance of approximately 20%, the sample size was estimated as 1537[20].

Data Collection tool

A 26-item self-administered questionnaire was used for this study. Content validation was carried out by three lecturers of the Department of Clinical Pharmacy and Pharmacy Management, Faculty of Pharmaceutical Sciences, University of Nigeria Nsukka. Section A of the questionnaire had eight items on the sociodemographic characteristics of the participants. Section B contained eight questions for assessing the knowledge of the participants regarding the COVID-19 vaccine with a "Yes" or "No" response option. Section C consisted of eight perception items regarding the COVID-19 vaccine with a five-point Likert scale response from Strongly disagree- Strongly agree. Finally, two questions were used to assess the Acceptance level of the participants towards the COVID-19 vaccine with a "Yes" or "No" or "I don't know" response option. The questionnaire was distributed to the students in various settings such as their hostels, in their classrooms before lectures, or at public spaces within the institution's premises.

Data Analysis

The filled questionnaires were coded and entered in Microsoft Excel. Afterwards, the raw data was cleaned and then exported into the SPSS (Statistical Products and Service Solution) version 25.0 for analysis. Frequencies and percentages were used to describe the analysed data. Inferential statistics were done by computing the Chi-square test of association between knowledge, perception and acceptance scores and the demographic characteristics of the respondents and the p-value was set as 0.05.

RESULTS

In Table 1, 1,143 students completed the survey while the estimated sample size was 1537. Therefore, the response rate was 74%. Most of them, 814 (71.2%), were within the age range of 18-24 years. Slightly more than half 610 (53.4%) of the students live off-campus. Most of the

students, 1054 (92.2%) gained admission through the post-Unified Tertiary Matriculation Examination (UTME). A total of 577 (50.5%) of the respondents had a good knowledge of the COVID-19 vaccine as seen in Figure 1. In Table 2, the statement "Is it dangerous to use an overdose of COVID-19 vaccine?" had the highest percentage of "Yes" answers 1031 (90.2%). Also, 585 (51.2%) of the respondents stated "No" to the statement "Is COVID-19 vaccine suitable for pregnant women?" From the study, 94.0% had not taken the COVID-19 vaccine. The level of acceptance was very low, 69 (6%), and only about 5% of those who had taken it would recommend it to family or friends. In Table 3, almost half of 478 (41.9%) agreed that there are "Scary information about the vaccine is rampant on social media". Also, 454 (39.7%) of the respondents agreed that "COVID-19 vaccine is safe and essential for use". Table 4 shows that there was no significant association (p >0.05) between the sociodemographic variables and the knowledge about the COVID-19 vaccine. However, the perception of the respondents towards the COVID-19 vaccine was significantly associated with gender (p = 0.010) and institution (p <0.001). In addition, knowledge and perception of the COVID-19 vaccine were significantly associated with its acceptance at p= 0.038 and < 0.001, respectively.

Discussion

This study's findings evaluated the level of knowledge, perception, and acceptance of the COVID-19 vaccine among undergraduate students in three higher institutions in Enugu state, Nigeria. Most of the students gained admission through the post Unified Tertiary Matriculation Examination (UTME). It is not surprising as studies have shown that post-UTME is the most popular mode of entry into universities for undergraduate students[21,22]. The post-unified Tertiary Matriculation Examination is a computer-based standardised test developed by the Joint Admissions and Matriculation Board (JAMB). The purpose of the examination is for admissions to undergraduate programs of tertiary institutions in Nigeria. About half of the students had a good knowledge and

perception towards the use of the COVID-19 vaccine. One may assume that such an observation was because undergraduate students are expected to be exposed to certain information inaccessible to the other less privileged members of the general population. In this study, gender and institution of study were significantly associated with the perception of undergraduate students towards the COVID-19 vaccine. It was also observed that female students had better knowledge, perception, and acceptance of the COVID-19 vaccine than their male counterparts.

The modal age category for this study was 18-24 years. This is comparable with a study on university students in Egypt which stated that the mean age of respondents was 20.51 ± 1.66[23]. However, a study on Nigerian university students reported that the majority of the students were in the age group of 18-27 years[24] and another study showed a modal age but of a wider range; 18-35 years[25]. Gender was found to be statistically associated with the perception of the COVID-19 vaccine among the participants in this current study unlike what was obtained in the study by Orok et at[24]. The fact that the majority of the respondents were females may have contributed to this association. Most of the respondents were females which is consistent with an online population study carried out in Malaysia[17] and a study among West African countries[26].

The students had a good knowledge of the COVID-19 vaccines, but a study carried out by Jiang et al also discovered good knowledge about the COVID-19 vaccine among the participants[27]. Positive perception towards COVID-19 vaccination among foreign medical students in China was found to be as high as the observed level in the current study. A good proportion of the respondents were convinced that the COVID-19 vaccines are safe and effective. This is comparable with the findings from the study by Jiang and colleagues[27]. However, the percentage of those who think it is effective is much lower than those in the study of Mose and his team of researchers[10]. The reason could be that their study was done exclusively among medical and health science students.

Acceptance of the COVID-19 vaccine by the students was associated with their knowledge and perception of the COVID-19 vaccine. This is in line with the outcomes from a scoping review done on COVID-19 vaccine hesitancy in Africa. It was found that among other factors that facilitate the acceptability of the COVID-19 vaccine were knowledge and positive perception about the vaccine[6]. Like our study, gender is seen to be significantly associated with the perception of the students towards the COVID-19 vaccine.

Conclusion

University students in Enugu State, Nigeria had a good knowledge and positive perception towards COVID-19 vaccination but there is still a need to increase their knowledge to dispel the myths about vaccination.

Author contributions

Conceptualization: [AUM, BOU-K]
Methodology: [AUM, AE, BOU-K]
Formal analysis and investigation: [AUM, AE, EJU, BOU-K];
Writing - original draft preparation: [AE, BOU-K];
Writing - review and editing: [AUM, AE, EJU, BOU-K];
Resources: [AUM, BOU-K];
Supervision: [BOU-K]

Policy Implication

There is a need to ensure that information concerning the COVID-19 vaccine should be spread to all institutions via health promotion campaigns and public health talks. The heads of tertiary institutions should organise seminars, workshops on vaccination acceptance and recommendations to all students.

Funding information

This research received no specific grant from any funding agency in the public, commercial, or not-for-profit sectors.

Declaration of competing interest

The authors have no competing interests relevant to this article's content.

Acknowledgement

We acknowledge the students who participated in this study and the authors who helped write and edit this article.

REFERENCES

1. Wiersinga W, Rhodes A, Cheng AC, Peacock SJ. Pathophysiology, Transmission, Diagnosis, and Treatment of Coronavirus Disease 2019 (COVID-19): A Review. *The Journal of the American Medical Association*. 2020;324(8):782-793. doi:doi:10.1001/JAMA.2020.12839

2. Parasher A. COVID-19: Current understanding of its Pathophysiology, Clinical presentation and Treatment. *Postgraduate Medical Journal*. 2021;97(1147):312-320. doi:10.1136/postgradmedj-2020-138577

3. Sanyaolu A, Okorie C, Marinkovic A, et al. Comorbidity and its Impact on Patients with COVID-19. *SN Compr Clin Med*. 2020;2(8):1069-1076.

4. WHO. Weekly epidemiological update on COVID-19 - 29 June 2021. Published 2021. Accessed October 29, 2023. https://www.who.int/publications/m/item/weekly-epidemiological-update-on-covid-19---29-june-2021

5. WHO. Coronavirus Disease (COVID-19) Dashboard With Vaccination Data. Published 2023. Accessed May 26, 2023. https://covid19.who.int/region/afro/country/ng

6. Ackah BBB, Woo M, Stallwood L, et al. COVID-19 vaccine hesitancy in Africa: a scoping review. *Global Health Research and Policy*. 2022;7(1):1-20. doi:10.1186/s41256-022-00255-1

7. MacDonald NE, Eskola J, Liang X, et al. Vaccine hesitancy: Definition, scope and determinants. *Vaccine*. 2015;33(34):4161-4164. doi:10.1016/j.vaccine.2015.04.036

8. Lazarus J V, Ratzan SC, Palayew A, et al. A global survey of potential acceptance of a COVID-19 vaccine. *Nat Med*. 2021;27(2):225-228. doi:doi:10.1038/S41591-020-1124-9

9. Nzaji, Michel Kabamba Ngombe, Leon Kabamba Mwamba, Guillaume Ngoie Ndala DBB, Miema JM, Lungoyo CL, Mwimba BL, Bene ACM, Musenga EM. Acceptability of Vaccination Against COVID-19 Among Healthcare Workers in the Democratic Republic of the Congo. *Pragmatic and Observational Research*. 2020;11:103-109. doi:10.2147/POR.S271096

10. Mose A, Haile K, Timerga A. COVID-19 vaccine hesitancy among medical and health science students attending Wolkite University in Ethiopia. *PLoS ONE*. 2022;17(11):e026308. doi:10.1371/journal.pone.0263081

11. Kanyike, Andrew Marvin Olum R, Kajjimu J, Ojilong D, et al. Acceptance of the coronavirus disease-2019 vaccine among medical students in Uganda. *Trop Med Health*. 2021;49(1):1-11. doi:10.1186/S41182-021-00331-1

12. Mustapha, Mohammed Lawal, Basira Kankia Sha'aban A, Jatau, Abubakar Ibrahim Wada, Abubakar Sadiq Bala AA, Mustapha S, et al. Factors associated with acceptance of COVID-19 vaccine among University health sciences students in Northwest Nigeria. *PLoS ONE*. 2021;16(11):e0260672. doi:10.1371/JOURNAL.PONE.0260672

13. Zewude B, Habtegiorgis T. Willingness to take COVID-19 vaccine among people most at risk of exposure in Southern Ethiopia. *Pragmatic and Observational Research*. 2021;12:37-47. doi:10.2147/POR.S313991

14. Adebisi Y, Alaran A, Bolarinwa O, Akande-Sholabi W, Lucero-Prisno D. When it is available, will we take it? Social media users' perception of hypothetical covid-19 vaccine in Nigeria. *Pan Afr Med J*. 2021;38. doi:10.11604/PAMJ.2021.38.230.27325

15. Reuben R, Danladi M, Saleh D, Ejembi P. Knowledge, Attitudes and Practices Towards COVID-19: An Epidemiological Survey in North-Central Nigeria. *J Community Health*. 2021;46(3):457-470. doi:10.1007/S10900-020-00881-1

16. Jegede A. What led to the Nigerian boycott of the polio vaccination campaign? *PLoS Med*. 2007;4(3):417-422. doi:10.1371/.0040073

17. Mohamed N, Solehan H, Mohd Rani M, Ithnin M, Isahak C. Knowledge, acceptance and perception on COVID-19 vaccine among Malaysians: A web-based survey. 2021;16(8):e0256110. doi:10.1371/.pone.0256110

18. Sallam M, Dababseh D, Eid H, et al. High rates of COVID-19 vaccine hesitancy and its association with conspiracy beliefs: a study in Jordan and Kuwait among other Arab

countries. *Vaccines*. 2021;9(1):1-16. doi:10.3390/vaccines9010042

19. Vyas D, Galal S, Rogan E, Boyce E. Training students to address vaccine hesitancy and/or refusal. *American Journal of Pharmaceutical Education*. 2018;82(8):944-953. doi:10.5688/ajpe6338

20. Hajian-Tilaki K. Sample size estimation in epidemiologic studies. *Caspian Journal of Internal Medicine*. 2011;2(4):289-298.

21. Bello A, Abdullahi M, Ibrahim I, Muhammad IA. Comparative effect of mode of entry on undergraduates' achievement in science education programs in Federal university of Kashere, Gombe state, Nigeria. *Kashere Journal of Education (KJE)*. 2021;2(1):40-48.

22. Osoba M, Usman S, Oyadiran O, et al. Undergraduate medical education in nigeria: Current standard and the need for advancement. *Pan African Medical Journal*. 2021;40. doi:10.11604/pamj.2021.40.40.30542

23. Tharwat S, Saad A, Nassar M, Nassar D. Acceptance and hesitancy to receive COVID - 19 vaccine among university students in Egypt : a nationwide survey. *Trop Med Health*. 2023;51(16):1-12. doi:10.1186/s41182-023-00509-9

24. Orok E, Ndem E, Daniel E. Knowledge, attitude and perception of medical students on COVID-19 vaccines: A study carried out in a Nigerian University. *Frontiers in Public Health*. 2022;10. doi:10.3389/fpubh.2022.942283

25. Lee KW, Yap SF, Ong HT, Leong PP, Mohamad Hatta N, Lye MS. Knowledge, Perceptions and Behaviors Related to COVID-19 in a University Setting in Malaysia. *Frontiers in Public Health*. 2022;10(April):1-15. doi:10.3389/fpubh.2022.873022

26. Udoakang AJ, Djomkam ZA, Tapela K, et al. Knowledge, attitude and perception of West Africans towards COVID-19: a survey to inform public health intervention. *BMC Public Health*. 2022;22:1-12. doi:1186/s12889-022-12814-9

27. Jiang N, Gu P, Liu K, Song N JX. Acceptance of COVID-19 vaccines among college students: a study of the attitudes, knowledge, and willingness of students to vaccinate. *Hum Vaccines Immunother*. 2021;17(12):4914-4924. doi:10.1080/21645515.2021.2013077

Table 1: Demographic information of respondents (N=1,143)

Demographics		Number	Percentage
**Age (Years)**	Less than 18	51	4.5
	18-24	814	71.2
	25-31	269	23.4
	32-38	8	0.7
	Above 38	3	0.3
**Residence**	Hostel	533	46.6
	Off campus	610	53.4
**Gender**	Male	318	27.8
	Female	825	72.2
**Marital status**	Single	1074	94.0
	Married	69	6.0
**Mode of admission**	Post-UTME	1054	92.2

	Direct- Entry	89	7.8
**Religion**	Christianity	1114	97.5
	Islam	19	1.7
	African traditional religion	10	0.9
**Ethnic group**	Igbo	1079	94.4
	Yoruba	25	2.2
	Hausa	10	.9
	Cameroon	3	.3
	Annang	4	.3
	Ghanaian	2	.2
	Ibibio	7	.6
	Edo	4	.3
	Idoma	8	.7
	others	1	.1
**Higher institution**	University of Nigeria	538	47.1
	Institute of Management and Technology	279	24.4
	Enugu State University of Science and Technology	326	28.5

Table 2: Knowledge and Acceptance of COVID-19 Vaccine

S/N	Statements	No		Yes	
		Number	Percentage	Number	Percentage
1	Is COVID-19 vaccine given by only injection	228	19.9	915	80.1
2	Can COVID-19 vaccine protect the receiver from getting COVID-19 infection?	291	25.5	852	74.5
3	Is it dangerous to use an overdose of COVID-19 vaccine?	112	9.8	1031	90.2
4	Does COVID-19 vaccination give allergic reactions?	294	25.7	849	74.3
5	Does vaccination increase autoimmune disease?	365	31.9	778	68.1
6	Is COVID-19 vaccine suitable for pregnant women?	585	51.2	558	48.8
7	Can everyone including children receive the COVID-19 vaccine?	330	28.9	813	71.1
8	Do COVID-19 vaccines have side effects?	225	19.7	918	80.3
9	Have you taken the vaccine?	1074	94.0	69	6.0
10	If yes, do you encourage your family/friends/relatives to get vaccinated?	8	0.7	55	4.8

Table 3: Perception towards COVID-19 vaccine

S/N	Statements	Strongly Disagree		Disagree		Neutral		Agree		Strongly Agree	
		n	%	n	%	n	%	n	%	n	%
1	COVID-19 vaccine is safe	48	4.2	85	7.4	371	32.5	454	39.7	185	16.2
2	COVID-19 Vaccine is essential for use	66	5.8	125	10.9	347	30.4	454	39.7	151	13.2
3	COVID-19 may cause infection	128	11.2	406	35.5	362	31.7	160	14.0	87	7.6
4	COVID-19 vaccine may not be effective	89	7.8	188	16.4	341	29.8	415	36.3	110	9.6
5	It is not possible to reduce the incidence of COVID-19 without vaccination	156	13.6	247	21.6	241	21.1	330	28.9	169	14.8
6	Scary information about the vaccine is rampant on social media	22	1.9	49	4.3	139	12.2	478	41.8	455	39.8
7	COVID-19 vaccine can protect me from getting infected	117	10.2	150	13.1	394	34.5	330	28.9	152	13.3
8	I am afraid to take the COVID-19 vaccine	89	7.8	100	8.7	340	29.7	336	29.4	278	24.3

Table 4a: Association between Sociodemographic and level of Knowledge, Perception and Acceptance of the respondents

**Characteristics**		**Knowledge (A) N (%)**		**χ2**	**Perception (B) N (%)**		**χ2**	**Acceptance (C) N (%)**		χ2
		**Good knowledge**	**Poor Knowledge**		**Good Perception**	**Poor Perception**		**Acceptance**	**Rejection**	
**Age (Years)**	Less than 18	27 (2.4)	24(2.1)	1.522	29(2.5)	22(1.9)	3.760	3(0.3)	48(4.2)	9.115
P(A)= .823	18-24	415(36.3)	399(34.9)		481(42.1)	333(29.1)		47(4.1)	767(67.1)	
P(B)= .439	25-31	129(11.3)	138(12.1)		166(14.5)	101(8.8)		16(23.2)	251(22.0)	
P(C)= .058	32-38	5(0.4)	3(0.3)		6(0.5)	2(0.2)		2(0.2)	6(0.5)	
	Above 38	1(0.1)	2(0.2)		3(0.3)	0(0.0)		1(0.1)	2(0.2)	
**Residence**	Hostel	270(23.6)	263(23.0)	0.003*	321(28.1)	212(18.5)	0.017*	27(2.4)	506(44.3)	**1.355***
P(A)= .959*	Off campus	307(26.9)	303(26.5)		364(31.8)	246(21.5)		42(3.7)	568(49.7)	
P(B)= .897*										
P(C)= .244*										
**Gender**	Male	155(26.9)	163(28.8)	0.441*	171(15.0)	147(12.9)	**6.603***	21(1.8)	297(26.0)	0.130*
P(A)= .507*	Female	422(36.9)	403(35.3)		514(45.0)	311(27.2)		48(4.2)	777(68.0)	
P*(B)= .010										
P(C)= .718*										
**Marital Status**	Single	545(47.7)	529(46.3)	0.336*	642(56.2)	432(37.8)	0.085*	65(5.7)	1009(88.3)	<0.001*
P(A)= .562*	Married	32(2.8)	37(3.2)		43(3.8)	26(2.3)		4(0.3)	65(5.7)	
P(B)= .771*										
P(C)= 1.000*										

*Continuity correction value for 2 X 2 tables P(A). P(B) and P(C) stand for the p value for the knowledge, perception and acceptance of COVID-19 vaccine chi-square association with the respective variables.

Table 4b: Association between Sociodemographic and level of Knowledge, Perception and Acceptance of the respondents

Characteristics		Knowledge (A) N (%)		χ2	Perception (B) N (%)		χ2	Acceptance (C) N (%)		χ2
		**Good knowledge**	**Poor Knowledge**		**Good Perception**	**Poor Perception**		**Acceptance**	**Rejection**	
**Mode of Admission**	JAMB	540(47.2)	514(45.0)	2.690*	623(54.5)	431(37.7)	3.380*	65(5.7)	989(86.5)	.164*
P(A)= .101* *P*(B)= .066* *P*(C)= .686*	DE	37(3.2)	52(4.5)		62(5.4)	27(2.4)		4(0.3)	85(7.4)	
**Religion**	Christianity	564(49.8)	550(48.1)	1.723	666(58.3)	448(39.8)	1.917	65(5.7)	1049(91.8)	3.543
P(A)= .423	Islam	10(0.9)	9(0.8)		14(1.2)	5(0.4)		3(0.3)	16(1.4)	
P(B)= .383	ATR	3(0.3)	7(0.6)		5(0.4)	5(0.4)		1(0.1)	9(0.8)	
P(C)= .170										
**Ethnicity**	Igbo	548(47.9)	531(46.5)	10.742	651(57.0)	428(37.4)	11.907	62(89.0)	1017(89.0)	15.074
P(A)= .294	Yoruba	11(1.0)	14(1.2)		14(1.2)	11(1.0)		1(0.1)	24(2.1)	
P(B)= .219	Hausa	3(0.3)	7(0.6)		5(0.4)	5(0.4)		2(0.2)	8(0.7)	
P(C)= .089	Annang	2(0.2)	2(0.2)		3(0.3)	1(0.1)		0(0.0)	4(0.3)	
	Ghanaian	2(0.2)	0(0.0)		1(0.1)	1(0.1)		1(0.1)	1(0.1)	
	Ibibio	2(0.2)	5(0.4)		6(0.5)	1(0.1)		1(0.1)	6(0.5)	
	Edo	4(0.3)	0(0.0)		1(0.1)	3(0.3)		1(0.1)	3(0.3)	
	Idoma	4(0.3)	4(0.3)		3(0.3)	5(0.4)		1(0.1)	7(0.6)	
	Others	3(0.3)	3(0.3)		3(0.3)	2(0.2)		1(0.1)	5(0.5)	
**Higher Institution**	UNN	285(24.9)	253(22.1)	3.706	293(25.6)	245(21.4)	**22.422**	29(2.5)	509(44.5)	5.785
P(A)= .157	IMT	128(11.2)	151(13.2)		162(14.2)	117(10.2)		25(2.2)	254(22.2)	
***P*(B)= <.001**	ESUT	164(14.3)	162(14.2)		230(20.1)	96(8.4)		15(1.3)	311(27.2)	
P(C)= .055										
**Taken the Vaccine**	No	551 (48.2)	523 (45.8)	**4.283***	627(54.9)	447(39.1)	**16.749***	NA	NA	
P(A) = **< 0.038***	Yes	26 (2.3)	43 (3.8)		58(5.1)	11(1.0)				
P(B) = **< 0.001***										

NA: Not applicable because taking the vaccine was interpreted as acceptance

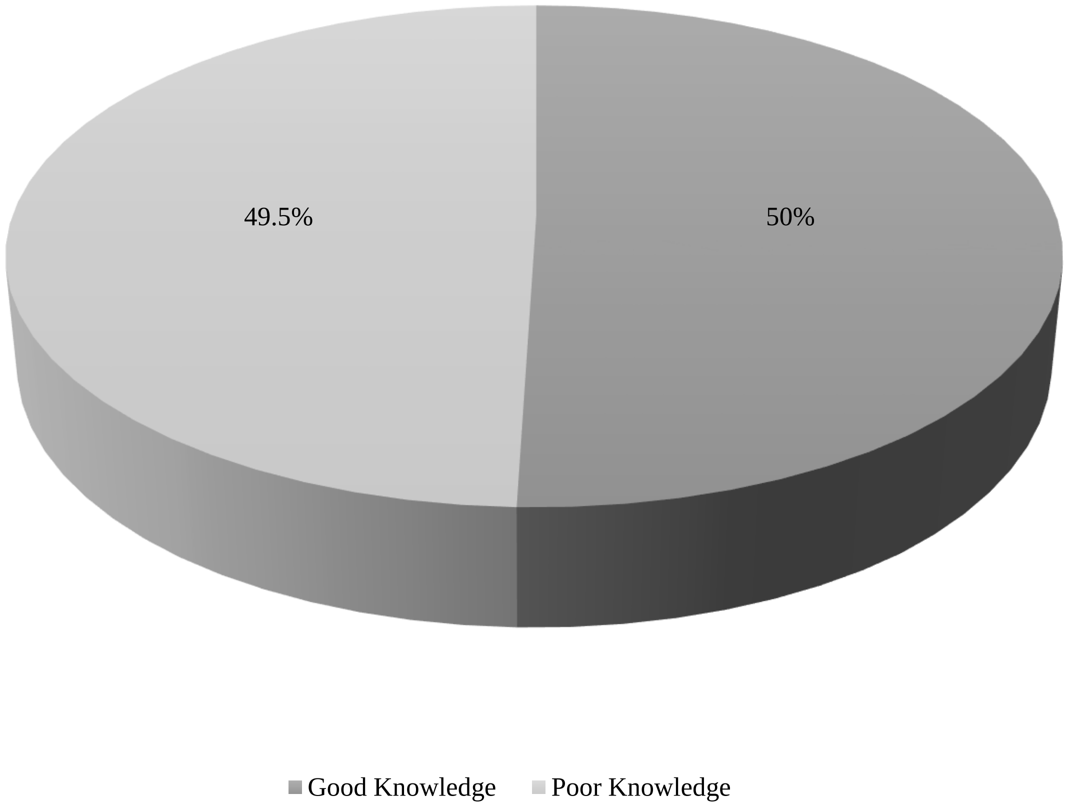

Figure 1; The level of Knowledge of COVID-19 Vaccine among University Students

Click here to access/download
Supporting Information
Supplementary material - Study questionnaire.docx
