# Supplementary material for: Factors associated with post-pandemic acceptance of COVID-19 vaccines among students in three Nigerian universities
Source: PLoS One. 2024 Dec 4;19(12):e0312271. doi: 10.1371/journal.pone.0312271 (PMC11616807; doi:10.1371/journal.pone.0312271)
Supplement: Attachment — Submitted filename: RESPONSE TO REVIEWERS.pdf [file pone.0312271.s003.pdf]

School of Medicine,
University of Nottingham,
Nottingham,
United Kingdom.
July 31, 2024.

The Editor-in-Chief,
PLOS ONE.

Dear Sir,

A point-by-point response to Reviewers' comments

Please find below our response to the points raised by the reviewers.

Best wishes,

Blessing Onyinye Ukoha-Kalu; *BPharm, MPharm, PharmD, PhD, FHEA*

Title: "Factors associated with post-pandemic acceptance of COVID-19 vaccines among students in three Nigerian Universities"

RESPONSE TO REVIEWERS

REVIEWER 1

1. **The authors need to review other local studies on this subject and situate them in the background to the study and discussion.**

   Thank you for your review and the suggestions of local studies to be included in the background and discussion. We have incorporated them on pages …

2. **What constitutes good knowledge and perception needs to be outlined.**

   The respondents with scores equal or more than the median score have good knowledge while those having scores equal or above the median are categorized as having a positive perception. This statement has been inserted in the revised version of the manuscript.

3. **The p-values in the tables should be placed in a separate column, not under the characteristics. Percentages of teh gender under knowledge added up to 1279%. It is not clear hwy this should be so.**

We have corrected the observations. Actually we made use of the percentages within each category of the variables to give a true picture of the proportions.

4. **Finally, the discussion needs depth. Results from this study and other referenced studies were merely quoted with little attempt to critically appraise the findings and situate them within the context of this study and referenced works. Feeble attempt was made to provide plausible explanation for most findings from this study.**

   The discussion has been rewritten considering your comments above. A critical appraisal and plausible explanation of most findings from the study were made within the context of the study and referenced works.

5. **The authors have left out the main finding form this study, low COVID-19 uptake. Modifiable factors that affect this needs to be mentioned, as a way of recommendation.**

   We have commented in the conclusion of the manuscript the need to modify the influencing factors that may increase the uptake of vaccine.

6. Few edits and comments are made in the attached manuscript.

   The edits were accepted while other minor corrections were effected in the revised version of the manuscript.

REVIEWER 2

Introduction
Suggestions
1. Streamline the introduction to focus on the study's objective and the specific context of university students in Enugu state, Nigeria.

Thank you for this suggestion. We have tried to focus more on the study’s objective.

2. Summarize or omit some statistics to maintain a clear and concise narrative.

We have expunged some statistics as well as made the narrative concise.

3. Provide a clearer transition between the background information and the study's objective.

We have provided a clearer transition between the background information and the study's objective.

4. Identify a specific research gap that the study aims to address, and explain how the findings will contribute to the existing literature.

We have identified a specific research gap that the study aims to address and also explained how the findings will contribute to the existing literature.

Results

I would recommend to Include additional variables, such as socioeconomic status, religious beliefs, and access to healthcare, to gain a more comprehensive understanding of the factors influencing vaccination decisions and to consider using more advanced statistical analysis techniques, such as logistic regression, to identify predictors of COVID-19 vaccine acceptance.

Thank you very much for this suggestion. Unfortunately, we would not be able to incorporate them. However, we shall make reference to them under limitations of the study.

Method

some improvements could be made to increase the validity and generalizability of the study findings.

Thank you for the suggestions. There was an error in the method section. We meant to say face validation and not content validation. This will be included in the limitations of the study. There was no content validation analysis.

Discussion

However, the discussion section could have demonstrated more critical thinking, exploring potential biases and limitations in the study and include clearer implications for policy, practice, or future research. Also include the potential impact of the study's findings on public health initiatives and vaccination campaigns.

We have tried to write the discussion section demonstrating more critical thinking and exploring potential biases and limitations in the study as well as clearer implications for policy, practice or future research.

REVIEWER 3

1. Check Reference number 9.
2. The modal age category for this study was 18-24 years on what basis?

1. Please, It is not clear what you wanted us to check about reference number 9.

2. The modal age category meant that majority of the respondents fell within this age range. However, since previous reviewers have queried the relevance of this to the objectives of the study, we have expunged it from the discussion section.
