# Supplementary material for: Factors associated with post-pandemic acceptance of COVID-19 vaccines among students in three Nigerian universities
Source: PLoS One. 2024 Dec 4;19(12):e0312271. doi: 10.1371/journal.pone.0312271 (PMC11616807; doi:10.1371/journal.pone.0312271)
Supplement: Attachment — Submitted filename: RESPONSE TO REVIEWERS.pdf [file pone.0312271.s004.pdf]

School of Medicine,
University of Nottingham,
Nottingham,
United Kingdom.
August 28, 2024.

The Editor-in-Chief,
PLOS ONE.

Dear Sir,

<u>A point-by-point response to Reviewers' comments</u>

Please find below our response to the points raised by the reviewers.

Best wishes,

Blessing Onyinye Ukoha-Kalu; *BPharm, MPharm, PharmD, PhD, FHEA*

Title: "Factors associated with post-pandemic acceptance of COVID-19 vaccines among students in three Nigerian Universities"

RESPONSE TO REVIEWERS

REVIEWER 1

1. Provide more justification for selecting three institutions from the possible 30 in Enugu State. Please clarify the sampling technique used and the selection criteria.

Thank you for your comment. We have now provided more justification for selecting three institutions. Please see page 4:

"…These institutions were chosen based on their large student populations, diverse academic offerings, and significant geographical coverage within the state. We prioritized institutions with large and diverse student bodies to ensure that our sample would be representative of the broader student population in Enugu State. To capture a range of perspectives and experiences, we selected institutions that differ in their academic focus and type (a federal university, a state university, and a polytechnic). The selected institutions are strategically located in different parts of Enugu State, ensuring that the study captures data from students in various urban and rural settings…

… This study employed a non-probability purposive and convenience sampling technique to recruit the participants."

2. Revise the conclusion to focus on the most important results and their significance. Clearly state the study's contributions, implications, and provide a final thought or call to action (around 100-150 words).

Thank you for your comment. We have now revised the conclusion to focus the most important results and their significance. Please see page 8:

"This study provides valuable insights into the post-pandemic acceptance of COVID-19 vaccines among university students in Enugu State, Nigeria. Despite a moderate level of knowledge and generally positive perceptions towards the vaccine, acceptance rates remained low, influenced significantly by the students' perceptions rather than their knowledge alone. The findings underscore the importance of targeted educational interventions that not only disseminate accurate information but also actively address misconceptions and enhance positive perceptions. This approach could be instrumental in improving vaccine uptake among this demographic. Our study highlights the critical role of perception in vaccine acceptance, suggesting that future public health campaigns should prioritize shifting perceptions through culturally sensitive, institution-specific strategies. These findings contribute to the ongoing dialogue on vaccine hesitancy and suggest actionable steps for increasing vaccine acceptance in similar contexts."

3. Proofread the manuscript to correct spelling errors, ensure clarity and precision in your writing.

Thank you for your comment. We have now proofread the manuscript using an English editing service.
